# MALAT1 promoted invasiveness of gastric adenocarcinoma

**DOI:** 10.1186/s12885-016-2988-4

**Published:** 2017-01-11

**Authors:** Na Keum Lee, Jung Hwa Lee, Cristina Ivan, Hui Ling, Xinna Zhang, Chan Hyuk Park, George A. Calin, Sang Kil Lee

**Affiliations:** 1 Division of Gastroenterology, Department of Internal Medicine, Institute of Gastroenterology, Yonsei University College of Medicine, 50 Yonsei-ro, Seodaemun-gu, Seoul, 120-752 Republic of Korea; 2 Department of Experimental Therapeutics and Center for RNA Interference and Non-Coding RNAs, University of Texas M. D. Anderson Cancer Center, Houston, TX USA

**Keywords:** MALAT1, Gastric cancer, Invasion, Metastasis, Apoptosis

## Abstract

**Background:**

Gastric cancer is the second leading cause of cancer globally, and the mechanism of its pathogenesis is still largely unknown. Recently, non-coding RNAs have been recognized to promote metastasis in various cancers, including gastric cancer.

**Methods:**

We found that metastasis associated lung adenocarcinoma transcript-1 (MALAT1) is upregulated in gastric cancer tissue compared to adjacent normal tissue, as determined by microarray and subsequent qRT-PCR, then investigated the impact of MALAT1 on apoptosis, cell proliferation, and the cell cycle to dissect the carcinogenesis of gastric cancer, and examined mechanisms of invasion and metastasis. Expression of MALAT1 and U6 was determined by SYBR qRT-PCR in nine-teen gastric cancer cell lines and fifty fresh samples of cancer tissue and adjacent tissues. Downregulation of MALAT1 was accomplished with two different siRNAs. Cell proliferation was determined after treatment with these siRNAs. FACS using PI/Annexin-V staining was carried out. To analyze the invasiveness, a scratch wound-healing assay and a Matrigel invasion assay were performed. Cancer related gene expression assay was done after transfection of siR- MALAT1.

**Results:**

The expression of MALAT1 was significantly elevated in various gastric cancer cell lines and gastric cancer tissues compared to normal cell lines and tissues (*p <* 0.01). siR-MALAT1 significantly reduced viable AGS cell numbers and induced apoptosis (*p* < 0.05). Deep invasion of tumor (advanced T stages) was more common in the high MALAT1-level group (*p* = 0.039). siR-MALAT1 significantly decreased AGS cell invasiveness and migration. siR-MALAT1 reduced expression of snail and N-cadherin, and elevated E-cadherin. The Wnt/β-catenin related genes were significantly decreased by transfection of siRNA MALAT1. MALAT1 is involved in gastric carcinogenesis via inhibition of apoptosis and promotes invasiveness via the epithelial-to-mesenchymal transition.

**Conclusions:**

In our study, we found that deregulation of MALAT1 could be involved in both tumorigenesis and invasiveness in gastric cancer cells.

**Electronic supplementary material:**

The online version of this article (doi:10.1186/s12885-016-2988-4) contains supplementary material, which is available to authorized users.

## Background

Gastric cancer is one of the major causes of death worldwide; however, the mechanism of development and progression of gastric cancer is largely unknown [1, 2]. Recent studies have revealed that non-coding RNAs such as microRNAs regulate epigenetic gene expression and are dysregulated in some gastric cancers [3–6].

Long non-coding RNAs (lncRNAs) are a newly-defined class of ncRNA with lengths greater than 200 nucleotides, and play important roles in biological processes [5]. To date, some lncRNAs are known to be involved in carcinogenesis and metastasis of various cancers [3, 7–10]. We previously reported that HOTAIR can regulate invasion and cell proliferation in gastric cancer [11]. In consequence of this finding, we speculated that there might be more lncRNAs involved in gastric cancer development. lncRNA expression profiles of certain diseases have been identified by microarray and RNA seq [12, 13].

Metastasis associated lung adenocarcinoma transcript-1 (MALAT1) is known to be involved in alternative splicing of pre-mRNAs by cell- or tissue-type-specifically modulating serine/arginine (SR) splicing factors [14, 15]. In particular, MALAT1 (~8 kb) in the form of nuclear-retained regulatory RNAs (nrRNAs) acts by interacting with SR proteins and regulating their cellular level in nuclear speckle domains in a phosphorylation-dependent manner [16]. MALAT1 is significantly more highly expressed in non–small cell lung carcinoma (NSCLC) patients and induces invasion, migration, and tumor growth in many cancer types, including lung cancer, uterine endometrial stromal sarcoma, colorectal cancer, and hepatocellular carcinoma [17–21]. However, MALAT1 in gastric cancer has not been studied as of yet, and functional and mechanistic studies of MALAT1 are inadequate and unclear [1].

In this study, we found changes in the expression of MALAT1 in adjacent gastric normal and cancer tissues through microarrays. Based on the microarray analysis, we evaluated the impact of MALAT1 on apoptosis and cell proliferation as indicators of carcinogenesis in gastric cancer. We also investigated the clinical significance of MALAT1 level as a predictor of severity of clinicopathological factors in patients with gastric cancer, and tried to dissect MALAT1’s molecular mechanisms with respect to invasion and metastasis in vitro.

## Methods

### Patients and tissue samples

Fifty fresh gastric cancer tissue and paired adjacent gastric tissue samples were obtained from 50 patients who underwent surgical resection for gastric cancer at Severance Hospital, Yonsei University College of Medicine. All samples were frozen in liquid nitrogen immediately after resection and stored at −80 °C until use. The mean age of patients was 60.7 (39–79) years and the male:female ratio was 2.2:1.

### Cell lines and cell culture

A total of 22 gastric cancer cell lines was used. The Yonsei Cancer Center (YCC) series had obtained from Song-dang Institute for Cancer Research, Yonsei University College of Medicine. Cell lines were obtained from the Korean Cell Line Bank (KCLB, SNU, Seoul, Korea) and the American Type Culture Collection (ATCC, Rockville, MD, USA). MKN 28, MKN 74, and AGS were cultured in RPMI-1640 medium (Thermo Scientific, Rockford, IL, USA) supplemented with 10% fetal bovine serum (FBS) and 1% penicillin and streptomycin solution. The cells were maintained in a humidified atmosphere of 5% CO_2_ and 95% air at 37 °C.

### Microarray and data analyses

New ncRNA microarray platforms from The University of Texas MD Anderson Cancer Center that are not commercially available were used in this study. This array contains a collection of probes for various types of non-coding RNAs (18,669 probes corresponding to 1338 human pre-miRNAs, 8980 probes corresponding to 660 mouse pre-miRNAs, 3320 probes corresponding to 484 ultraconserved genes, 16,314 pyknon probes, and 2275 probes corresponding to 192 other non-coding RNAs). LncRNA Expression profiling analysis of the 3 paired samples including gastric cancer tissues and their matched adjacent normal tissues was performed using an in-house constructed microarray. Clinical characteristics of the three gastric cancer patients included for non-coding microarray are as follows: Two male and one female, each histological grade are well-differentiated, moderately-differentiated, and poorly differentiated adenocarcinoma. Their stages at diagnosis were IB, IIB, and IIIA, respectively.

Bioinformatic analysis was performed using R (version 3.0.1) (http://www.r-project.org) and Bioconductor (http://www.bioconductor.org/). The raw intensity for each probe is the median feature pixel intensity with the median background subtracted. Setting an offset of 1 ensures that there will be no negative values after log-transforming data. Data was quantile normalized followed by log_2_ transformation. Signals from probes measuring the same ncRNA were averaged. A linear model was fitted to each gene and empirical Bayes methods were used to obtain statistics. Statistical significance was defined as a *p*-value less than 0.05. Probes differentially expressed between samples were thus identified. The analysis was performed using the functions of the LIMMA library. Heatmaps were generated using the heatplot function of the made4 library.

### NanoString nCounter gene expression analysis

A total of 730 cancer-related human genes and 40 internal reference genes were contained on cancer pathway panel reporter and capture probe. For analysis, MKN28 cells were transfected with siCT or siMALAT1. 100 ng of Total RNA was used for hybridization, and analyzed on an nCounter Digital Analyzer (NanoString Technologies, Inc., Seattle, WA) according to the manufacturer’s instructions. Data quality control was implied using nSolver analysis software (NanoString Technologies). Reporter counts were normalized to each sample using positive control and housekeeping genes. The mean values were shown to fold change or log transformation.

### Small interfering RNA (siRNA) transfection

RNA interference-mediated knockdown of MALAT1 was carried out using target siRNAs for MALAT1. MKN28, MKN74, and AGS cells were transfected with three different siRNAs for MALAT1 or stealth siRNA (Invitrogen, Carlsbad, CA, USA) using Lipofectamine 2000 (Invitrogen). Target sequences for siRNAs targeting MALAT1 were: siMALAT1-1, sense: 5’-GAUCCAUAAUCGGUUUCAA-3’, antisense: 5’-UUGAAACCGAUUAUGGAUC-3’; siMALAT1-2, sense: 5’-CACAGGGAAAGCGAGUGGUUGGUAA-3’, antisense: 5’-UUACCAACCACUCGCUUUCCCUGUG-3’; and MALAT1-3, sense: 5’-GCAGAGGCAUUUCAUCCUU-3’, antisense: 5’-AAGGAUGAAAUGCCUCUGC-3’.

### Total RNA extraction, reverse transcription, and quantitative real-time PCR

Total RNA was isolated using TRIzol reagent (Invitrogen). The total RNA was reverse transcribed with Superscript II(Invitrogen) according to the manufacturer’s instructions. The expression of MALAT1 was determined with qRT-PCR using the SYBR Green method (Applied Biosystems Inc., Carlsbad, CA, USA). The level of MALAT1 was normalized to the internal control, U6. The Ct value was measured by the 2^-∆∆Ct^ method. The target sequences for MALAT1 and U6 were: MALAT1, Forward: 5’-ACTGAATCCACTTCTGTGTAGC-3’ and Reverse: 5’-CGGAAGTAATTCAAGATCAAGAG-3’; and U6, Forward: 5’-CTCGCTTCGGCAGCACA-3’ and Reverse: 5’-AACGCTTCAGGAATTTGCGT-3’.

### Trans-well chamber-Matrigel invasion and migration assay

Invasion assays were performed with BD Biocoat trans-well chambers (BD Biosciences, San Jose, CA, USA) according to the manufacturer’s protocol. MKN74 and AGS cells were transfected with each siMALAT1 or siCT for 48 h. The transfected cells were then plated in the upper chamber of the trans-well chamber in RPMI 1640 Medium (Thermo Scientific) without fetal bovine serum (FBS). The bottom of the trans-well chamber was filled with RPMI 1640 with 10% FBS. The number of invading cells was counted on a bright-field microscope. Five portions of the membrane were randomly selected and the average value of invading cells was calculated. For the migration assay, transfected MKN74, MKN28, and AGS cells were replated. The cells were incubated until the formation of an approximately 80–90% confluent monolayer. A P-20 micropipette tip was then used to mimic a scratch wound. The ratio of migratory capacity was determined by measuring the width of the scratch at 0 and 24 h by bright-field microscopy.

### Western blotting

MKN74 and AGS cells were transfected with 100 nM siMALAT1 or siCT. The cells were lysed in lysis buffer. Cell lysates were resolved by 8–10% SDS-PAGE and transferred to PVDF membranes (GE Healthcare, Piscataway, NJ, USA). After transfer, membranes were incubated with primary antibodies in 5% BSA (Affymetrix, Inc. Santa Clara, CA, USA) for 24 h at 4 °C. The primary antibodies used were: mesenchymal marker N-cadherin (1:1000, BD Biosciences), snail (1:1000, Cell Signaling Technology), c-Myc (1:500, Santa Cruz), GSK-3β (1:200, Santa Cruz), β-actin (1:5000, Bioworld Technology, Louis Park, MN, USA) and β-catenin (1:500, Santa Cruz). To visualize the target proteins, probed blots were incubated in ECL solution (GenDEPOT, Barker, TX, USA) and exposed to an Image Quant LAS 4000 bio-molecular imager for 10 s to 5 min.

### Cell proliferation and apoptosis

AGS and MKN28 cells were transfected with each siMALAT1 or siCT and incubated in a time course ranging from 0 to 96 h. Cell proliferation was measured by CellTiter 96® AQueous One Solution Cell Proliferation Assay (MTS assay, Promega, Madison, WI, USA). At various time points, the cells were exposed to MTS reagent in the dark for 1 h. The extent of the reaction was quantified with a spectrophotometric plate reader set at 490 nm. Propidium iodide and fluorescein isothiocyanate (FITC)-Annexin V staining was carried out. The apoptotic ratio was determined by flow cytometry (BD Biosciences).

### Cell cycle analysis

MKN74 cells were transfected with siMALAT1 or siCT and washed with 1X PBS (Thermo Scientific). After washing, cells were fixed with 75% ethanol at −20 °C. for 24 h. The fixed cells were stained with propidium iodide (Sigma, St. Louis, MO, USA). Cell cycle profiles were analyzed via flow cytometry (BD Biosciences).

### Soft agar colony formation

For analysis tumorigenecity in vitro, base and top agarose were coated in 6-well cultured plates. 1.5 ml of 2X RPMI1640 containing 1% Ultrapure Low Melting Point Agarose (Gibco, Rockville, MD, USA) was added into each well as base. After solidification, the transfected cells were resuspended in 2X RPMI1640 UltraPure Low Melting Point Agarose as top. The plates were maintained in a humidified atmosphere of 5% in air at 37 °C for 3 weeks. Colonies were stained with 0.5% Crystal Violet and measured under bright field microscopy.

### Immunofluorescence

AGS cells were transfected with each siMALAT1 or siCT, and fixed with 4% paraformaldehyde for 20 min. After fixation, the cells were permeabilized with 0.3% Triton X-100 in PBS for 30 min, and blocked with 5% BSA in PBS for 1 h. After blocking, the cells were incubated in β -catenin (1:200, Santa Cruz) overnight at 4 °C. the cells were treated with Cy2-conjugated anti Mouse IgG (Jackson ImmunoResearch Laboratories) for 1 h in the dark. The cells were mounted with DAPI vecta shield (Vector Laboratories, Burlingame, CA, USA). The mounted cells were photographed using a Zeiss LSM 700 confocal microscope (Carl Zeiss, Oberkochen, Germany).

### Statistical analysis

All data analysis of continuous and categorical variables is presented as the mean ± standard error and the number of lesions with the percentage. Statistical tests used to compare the measured results included the *t*-test, *χ*2 test, and Fisher’s exact test. The degree of expression of MALAT1 in gastric cancer was categorized as low or high, based on the median of MALAT1 expression. Moreover, the Cox proportional hazard model was used to adjust for possible confounding variables, including sex, age, differentiation, and stage. A value of *p* < 0.05 was considered as a statistically significant difference for comparisons between groups. All statistical procedures were conducted using the statistical software SPSS for Windows (version 18.0; SPSS Inc., Chicago, IL, USA).

## Results

### Long non-coding RNA MALAT1 is significantly upregulated in gastric cancer

**Fig. 1:**
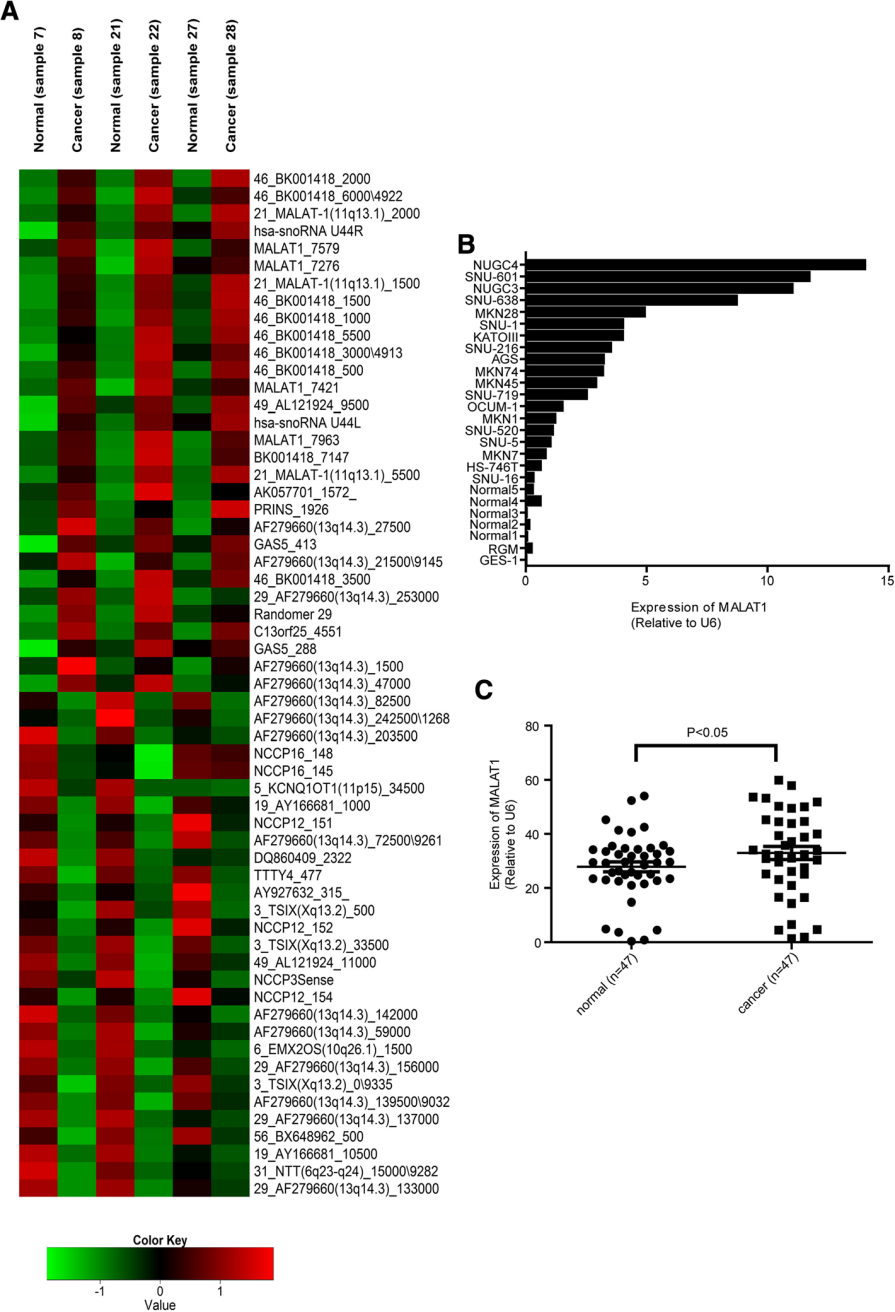
MALAT1 levels were measured in 47 of 50 patients with gastric cancer. Expression of MALAT1 was upregulated in gastric tissues and cell lines. Supervised microarray analysis was done with respect to 192 “other non-coding RNAs” to find previously annotated lncRNAs. Sample 7, sample 21, and sample 27 are paired with sample 8, sample 22, and sample 28, respectively. Up-regulated genes are shown in red and down-regulated genes are shown in blue (**a**). The expression of MALAT1 was measured in 19 gastric cancer cell lines, normal cell GES-1, RGM-1 and 5 normal adjacent tissues (**b**) and 47 fresh gastric cancer and paired adjacent gastric tissues (**c**). The expression of MALAT1 was normalized to U6. Data shown represent the mean ± s.e.m. Asterisks represent a statistically significant difference from the scrambled control (*P ≤ 0.05)

We analyzed the microarray results of the levels of 192 “other non-coding RNAs” to find previously annotated lncRNAs with differential expressional patterns between paired cancer and adjacent normal gastric tissues. Thirty probes were upregulated more than 2-fold in paired gastric cancer tissue relative to adjacent normal tissue, while 29 probes were downregulated more than 2-fold (Fig. 1a). Among these probes were examples such as GAS5, HOTAIR, and MALAT1, which have been reported to be involves in carcinogenesis. GAS5 has already been reported to be associated with the occurrence of gastric cancer [22], and HOTAIR has also been reported on by us [11]. We undertook to validate MALAT1, which has not been previously reported as an lncRNA involved in gastric adenocarcinoma.

### Altered expression of MALAT1 in gastric cancer cell lines and gastric cancer tissues

The level of MALAT1 was measured in 19 gastric cancer cell lines and compared to normal epithelial gastric GES-1, 5 normal gastric tissues adjacent to cancer and rat normal gastric surface mucous cell (RGM-1) (Fig. 1b). The expression of MALAT1 was upregulated in gastric cancer cell lines relative to levels in GES-1, 5 normal gastric tissues and RGM.

MALAT1 levels were measured in 47 of 50 patients with gastric cancer. The expression of MALAT1 was significantly higher in gastric cancer tissues than in adjacent normal tissues. (Fig. 1c) (*p* > 0.05). Clinicopathologic features were analyzed according to the level of MALAT-1 expression (Table 1). Patients with higher levels of MALAT-1 expression had greater depths of invasion than did those with low levels of MALAT-1 (*p* = 0.039). In addition, tumors of patients in the high-level MALAT-1 group tended toward more advanced TNM stage than did those in the low-level MALAT-1 group (high vs. low; stage III, 80.8% vs. 19.2%, *p* = 0.505), but this did not reach statistical significance. Patients with poorly differentiated histology or signet ring cell carcinoma had higher expression of MALAT1 compared to well to moderately differentiated adenocarcinoma (*p* = 0.030). Age and sex, were not related to MALAT-1 expression. We could not get good correlation between overall survival and expression level of MALT1 in this study (data not shown).

**Table 1:** The relationship between MALAT1 expression and clinicopathological feature of gastric cancer patients

Variables	Number	MALAT1 expression		*P*-value
		Low	High	
Age (years)				0.071
<65	24	3 (12.5)	21 (87.5)	
≥65	23	8 (34.8)	15 (65.2)	
Sex				0.725
Male	32	7 (21.9)	25 (78.1)	
Female	15	4 (26.7)	11 (73.3)	
Depth of tumor invasion				0.039
T1 ~ T2	10	5 (50.0)	5 (50.0)	
T3 ~ T4	37	6 (16.2)	31 (83.8)	
Lymph node metastasis				0.472
Absent	16	5 (31.3)	11 (68.8)	
Present	31	6 (19.4)	25 (80.6)	
Stage				0.505
I, II	21	6 (28.6)	15 (71.4)	
III	26	5 (19.2)	21 (80.8)	
Lauren’s classification				0.242
Intestinal	27	9 (33.3)	18 (66.7)	
Diffuse	18	2 (11.1)	16 (88.9)	
Mixed	2	0 (0.0)	2 (100.0)	
Differentiation				0.030
Well to moderate	25	9 (36.0)	16 (64.0)	
Poorly or signet ring cell	22	2 (9.1)	20 (90.9)	
Serum CEA value				>0.999
≤5	37	9 (24.3)	28 (75.7)	
>5	10	2 (20.0)	8 (80.0)	
Serum CA19-9 value				0.614
≤37	41	9 (22.0)	32 (78.0)	
>37	6	2 (33.3)	4 (66.7)	

### Inhibition of MALAT1 suppressed cell proliferation and induced apoptosis

**Fig. 2:**
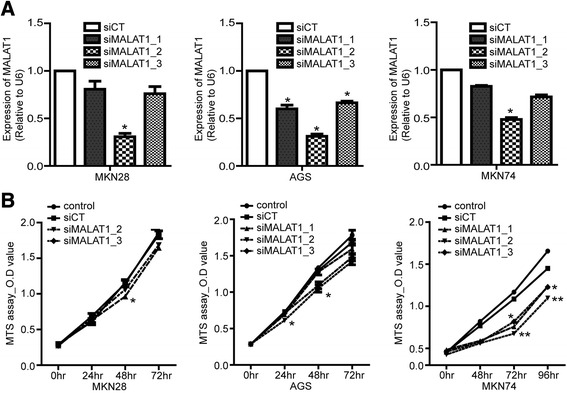
MALAT1 silencing suppressed cell proliferation. Gastric cancer cells were transfected with each siMALAT1 or siCT (**a**). Cell proliferation was analyzed by MTS assay (**b**). The data shown in panels are representative of three independent experiments. Data shown represent the mean ± s.e.m. Asterisks represent a statistically significant difference from the scrambled control (*P ≤ 0.05; **P ≤ 0.01)

We employed RNAi-mediated knockdown of MALAT1 in AGS, MKN74, and MKN28 cells to explore mechanisms of carcinogenesis. Three different siRNAs were tested to knock down MALAT-1 in three different gastric cancer cell lines (Fig. 2a). MALAT1 expression was decreased by transfection with siMALAT1 compared with levels in siCT-transfected cells. One of the MALAT1 siRNA’s (siMALAT1-2) consistently decreased the level of MALAT-1 to less than 50% of baseline in all three cell lines. Next we performed an MTS assay to check viable cell after transfection with siRNAs for MALAT1. In the AGS and MKN74 cell lines, the three different siRNAs significantly decreased the cell viability at both 24 and 72 h. The degree of reduction caused by siMALAT1-2 was greater than that by the other two siRNAs (Fig. 2b).

**Fig. 3:**
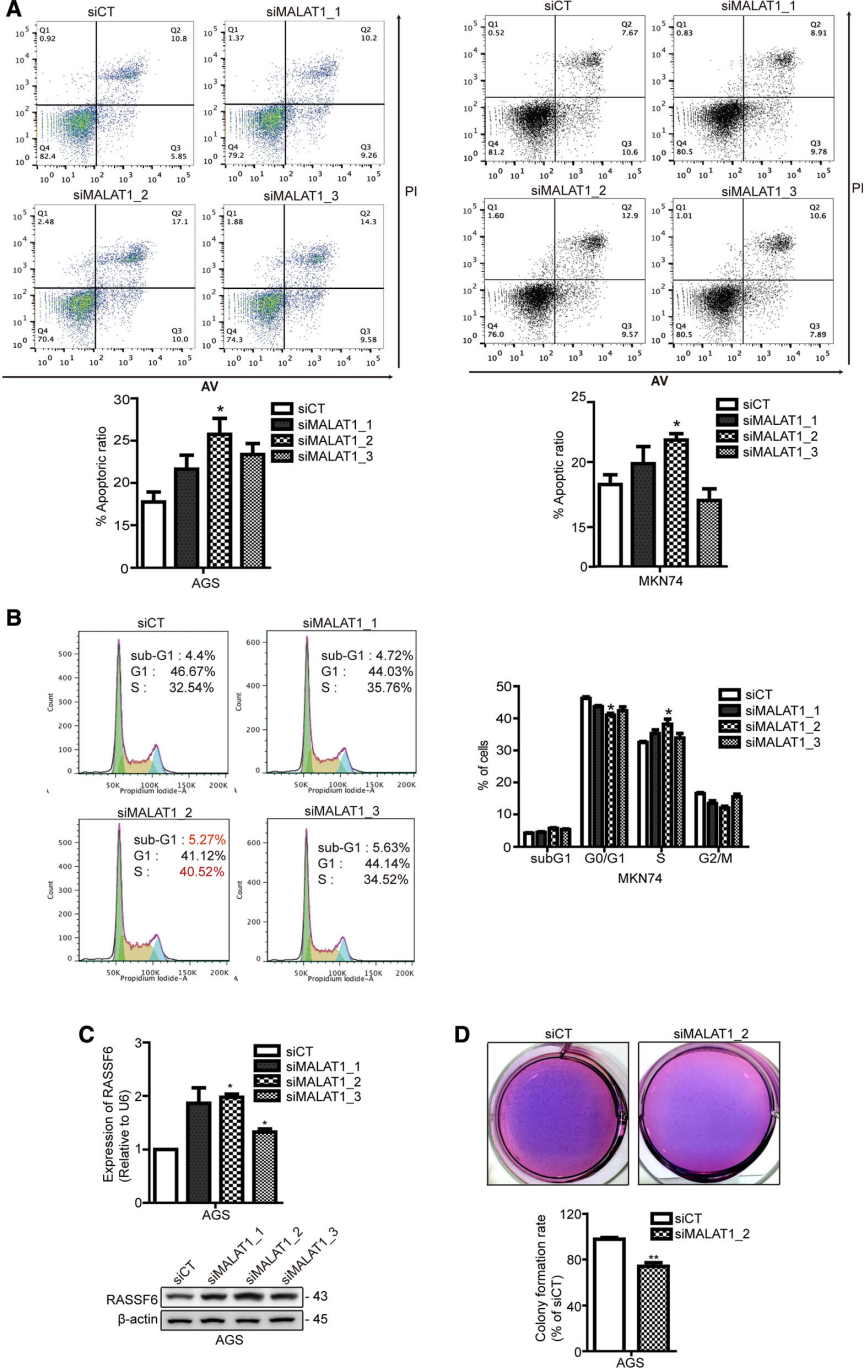
MALAT1 silencing induced apoptosis and cell cycle arrest. AGS and MKN74 cells were transfected with siMALAT1 or siCT, and apoptosis was detected by PI/Annexin V staining (**a**). Cell cycle arrest was analyzed by PI staining (**b**). The increased expression of RASSF6 by qRT-PCR and western blot (**c**). The decreased colony forming rate by siMALAT1_2 compared to siCT (**d**). The data shown in the panels are representative of three independent experiments. Data shown represent the mean ± s.e.m. Asterisks represent a statistically significant difference from the scrambled control (*P ≤ 0.05)

To analyze the effect of MALAT1 on cell proliferation, PI/Annexin V staining was carried out. The apoptotic ratio was significantly increased by siMALAT1-2 relative to siCT in MKN74 and AGS cells (Fig. 3a). However, knockdown of MALAT1 did not cause a significant difference in MKN28 cells (data not shown); this result indicates that certain phenotypic effects of MALAT1 may vary depending on the cell type.

### Inhibition of MALAT1 induces arrest in S phase and increased expression of RASSF6

As we found phenotypic effects in the form of cell death by assaying cell proliferation and apoptotic ratio, we then assayed defects in cell cycle progression, which can also be involved in apoptosis. The S phase population of cells was significantly increased in siMALAT1-2-transfected cells, compared to those transfected with siCT. The sub-G1 population, which is considered to represent apoptotic cells, was significantly increased by siMALAT1-2 transfection, compared to that in siCT-transfected cells, in the MKN74 cell line (Fig. 3b). When cell cycle progression was dysregulated by DNA damage, mutation and tumorigenesis can occur. This S phase arrest and increased sub-G1 population suggest that deregulation of MALAT1 may contribute to apoptosis via perturbations of cell cycle progression.

To explore the effect of MALAT-1 on apoptosis and cell cycle progression, we tested Ras association domain family (RASSF) 6, which is known to stabilize p53, regulate apoptosis and the cell cycle, and function as a tumor suppressor [23]. RASSF6 is also known to regulate the progression of gastric carcinogenesis [24]. The mRNA level of RASSF6 was significantly increased by treatment with three different siRNAs targeting MALAT1 compared with levels in the siCT-transfected group (Fig. 3c). The protein level of RASSF6 was also increased by treatment with siRNAs for MALAT-1 (Fig. 3c). This finding suggests that MALAT-1 may impair RASSF6’s function as a tumor suppressor, and thus promote gastric carcinogenesis. To monitor anchorage independent growth that measures cellular transformation, we performed soft agar colony formation in vitro. The clonogenic forming ability was decreased by siMALAT1_2-transfected cells compared to siCT-transfected cells (Fig. 3d). These results support that MALAT1 contributes to tumorigenesis of gastric cancer cell.

### Inhibition of MALAT1 decreased cell invasiveness and migration

**Fig. 4:**
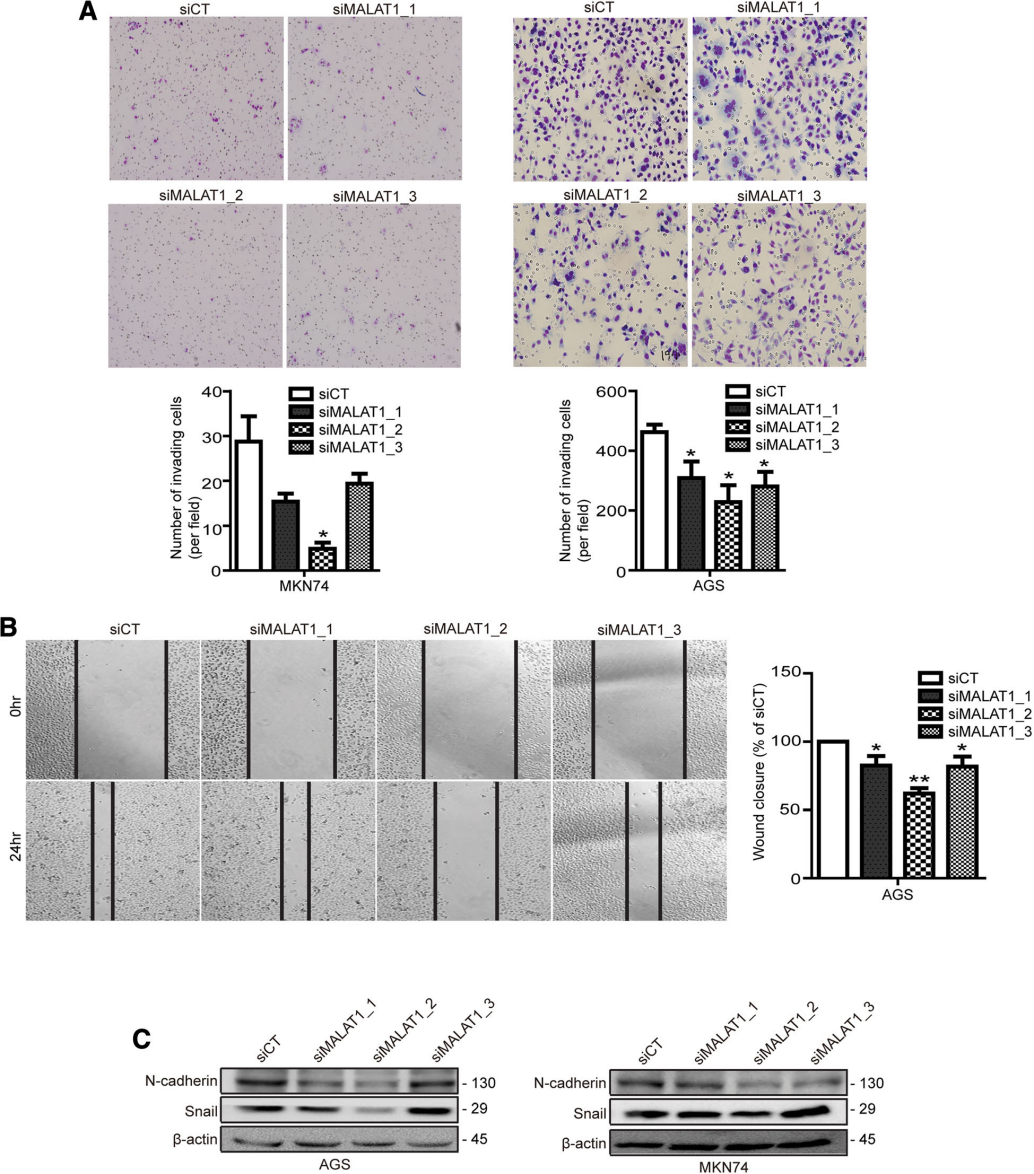
MALAT1 silencing inhibited cell invasiveness and the expression of EMT markers. AGS and MKN74 cells were transfected with siMALAT1 or siCT and cell invasiveness was measured by trans-well chamber assay (**a**). Wound-healing assays were performed (**b**). Levels of the EMT markers, N-cadherin and snail, were measured in AGS cells (**c**). Data are the mean of three independent experiments ± s.e.m. Asterisks represent a statistically significant difference from the scrambled control (*P ≤ 0.05; **P ≤ 0.01)

In our study, patients with high levels of MALAT1 had deeper invasion and more advanced stages than did patients with low expression of MALAT1. To determine the relationship between advanced stage and MALAT1, we explored the invasion and migration of gastric cancer cells using siRNA for MALAT1. In a trans-well chamber-Matrigel invasion assay, the invasion of MKN74 and AGS cells was significantly suppressed by all three siMALAT1s compared to that in the siCT-transfected group (Fig. 4a). A wound-healing assay was performed using the AGS cell line. All three siRNAs significantly reduced migration, which corresponded to the changes in the invasion assay (Fig. 4b).

To further investigate the changes in invasion and migration induced by siMALAT1s, we performed western blots using epithelial-to-mesenchymal transition (EMT) markers. The mesenchymal markers, snail and N-cadherin, were decreased by all three siMALAT1s compared to levels in the siCT-transfected group (Fig. 4c). In addition, expression of ZEB1, a mesenchymal marker, was decreased by siMALAT1s in AGS cells (data not shown).

### MALAT1 of high level is correlated with tumor invasion and MALAT1 silencing regulates β-catenin signaling

**Fig. 5:**
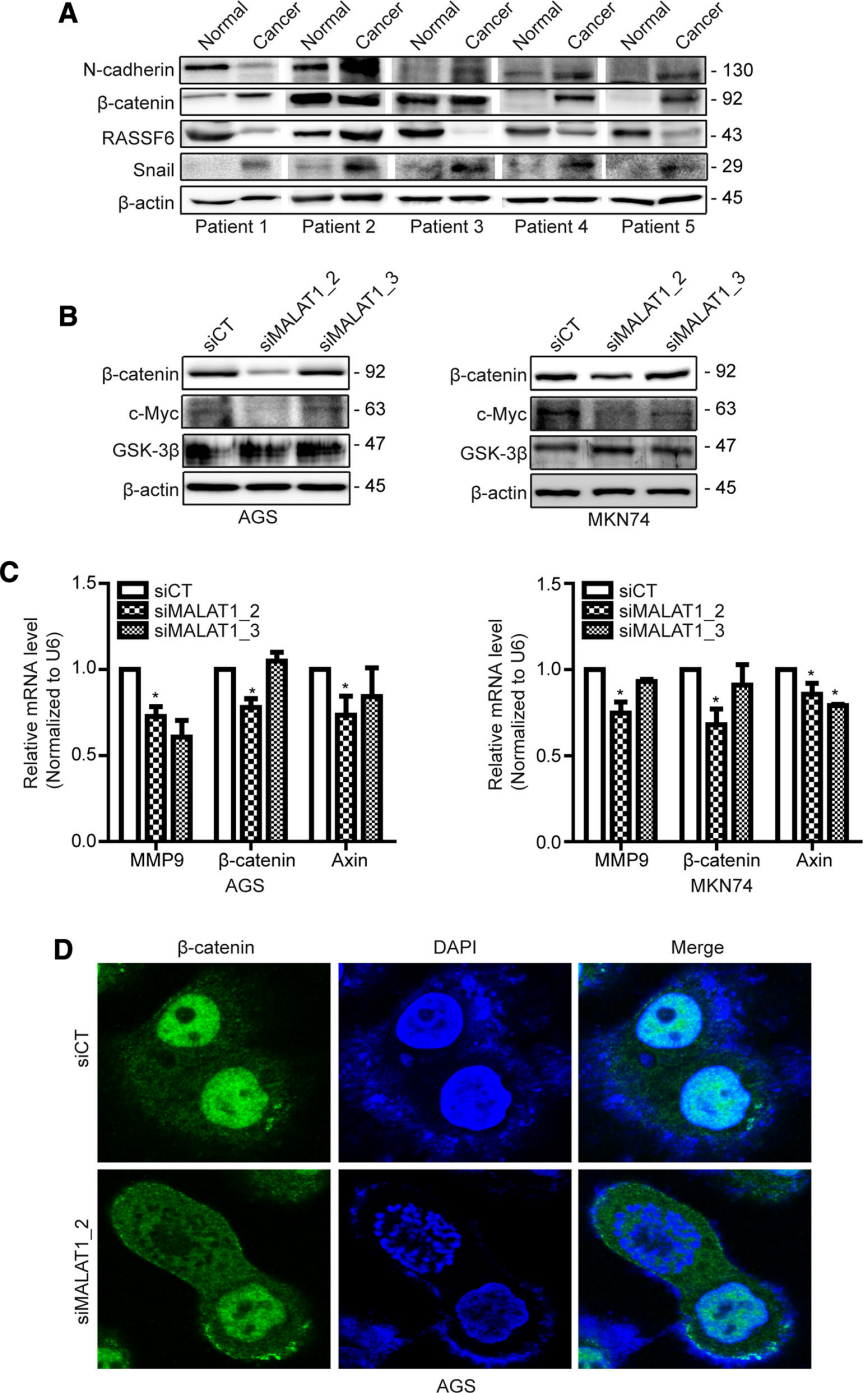
MALAT1 is involved in tumor invasion and regulates b-catenin related genes. In paired gastric cancer tissues form the 5 patients (patient #1 ~ #5), expression of MALAT1 and its target genes including N-cadherin, b-catenin, RASSF6 and Snail (**a**). AGS and MKN74 cells were transfected with siMALAT1s or siCT, and the expression of b-catenin signaling on western blot (**b**). Levels of the transcription factors, b-catenin, MMP9 and Axin were measured in AGS and MKN74 cells (**c**). Morphological change of b-catenin was determined by immunofluorescence after silencing of MALAT1 in AGS cells (**d**)

Based on our clinical data, we focused on the role of MALAT1 in tumor invasion. As the results, the expressions of N-cadherin, β-catenin and snail were generally elevated in cancer compared to adjacent normal tissues. Also, RASSF6 was shown decreased expression in cancer compared to adjacent normal tissues (Fig. 5a). Among 5 paired samples, snail, N-cadherin and β-catenin were high in cancer compared to adjacent normal tissue in 5, 4 and 3 patients, respectively. The level of RASSF6 was lower in cancer compared to adjacent normal tissue in 4 out of 5 patients.

In accordance with these results, we carried out NanoString gene expression array to investigate the mechanism. We found that expression of Wnt/beta-catenin signaling and its related-genes were significant affected by siMALAT1 compared to scramble (Supplement 1). CTNNB1 and WNT were markedly down-regulated by siMALAT1. The pattern of β-catenin and c-Myc were confirmed to be decreased by siMALAT1 compared to siCT in Western blot (Fig. 5b). The mRNA expression of MMP9, Axin and β-catenin also was significantly decreased in siMALAT1 compared to scramble in both AGS and MKN74 cells (Fig. 5c). According to these results, we analyzed the morphological changes of β-catenin by siMALAT1 in AGS cells using immunofluorescence. The nuclear localization of β-catenin was decreased by siMALAT1 compared to siCT (Fig. 5d).

## Discussion

It has been demonstrated that the long non-coding RNA MALAT1 may regulate many biological processes, including cancer development. We present here the novel finding that MALAT1 is increased in gastric cancer tissues compared to normal tissues according to microarray analysis. We confirmed that knockdown of MALAT1 induces apoptosis, arrest in S phase, and increased expression of the tumor suppressor, RASSF6. High expression of MALAT1 in gastric cancer correlated with advanced T stage, which was supported by the finding that Inhibition of MALAT1 decreased cell invasiveness and migration.

Microarrays are useful tools for global analysis of gene or protein content and expression. Recently a global expression profile of lncRNAs was analyzed by microarray technique [25]. We used house-made non-coding RNA expression arrays designed with probes targeting human pre-miRNAs, mouse pre-miRNAs, ultraconserved genes, pyknon probes, and other non-coding RNAs. We concentrated on 192 of these “other non-coding RNAs” to find annotated lncRNAs in this study. Analysis and validation of probes for UCR and pyknon will be conducted in a subsequent study. We identified 59 probes that were significantly differentially expressed between gastric cancer tissues and adjacent normal tissue. Of these, 30 lncRNAs were identified as consistently upregulated in all three HCC groups, while the other 29 lncRNAs were consistently downregulated. Among the upregulated lncRNAs in the microarray, we had already validated HOTAIR as having dysregulated expression in gastric cancer in a previous report [11].

MALAT1 is one of several well-known lncRNAs involved in many cancers, and our microarray results showed significantly differential expression level in gastric adjacent normal and cancer tissues. Based on these results, MALAT1 may play a crucial role in gastric carcinogenesis and metastasis, as do HOTAIR and GAS-5.

MALAT1 was already known to be correlated with cell proliferation and metastasis in a variety of cancers [19, 20, 26]. However, the functional role of MALAT1 in gastric cancer has not yet been studied. Wang J et al. reported that Serine/arginine-rich splicing factor 1 (SRSF1) downregulated Malat1 expression by enhancing RNA decay and inhibiting YAP activity in a promoter context-dependent manner in liver cancer [21]. We checked the relationship between SRSF1 and MALAT1 in gastric cancer cell lines (data not shown). We further explored whether MALAT1 could induce the constitutively active ∆Ron isoform by inducing alternative splicing of Ron, which is involved in cell dissociation, motility, and matrix invasion (data not shown). Next, we tried to elucidate how MALAT1 acts in gastric carcinogenesis through validation of some of its targets that are tumor suppressor genes. In particular, the metastasis-associated target gene, RASSF6, a member of the RAS-association domain family, is downregulated in gastric cancer, and its expression was upregulated by MALAT1 silencing, as revealed by genome-wide gene expression analysis [17, 24]. In our study, knockdown of MALAT1 induced increases in mRNA and protein levels of RASSF6 in AGS cells. It has previously been reported that expression of RASSF6 is correlated with invasion, lymph node metastasis, and advanced clinical stage in human gastric cancer [24].

Based on our results, expression of MALAT1 affects apoptosis and cell cycle progression, correlating with changes in cellular proliferation. Some of the gastric cancer cell lines, AGS and MKN74, showed significantly lessened cellular proliferation 48 h after treatment with siMALAT1, leading to an increased apoptotic ratio and S phase arrest corresponding with a decreased G0/G1 phase population. Wang J et al. reported an increased population in the G0/G1 phase of the cell cycle by propidium iodide staining. We observed an S phase arrest in MKN74 cells, unlike Wang J et al. These results indicated that even though the gastric cancer cells were conspecific, lncRNAs act at a specific time during development. Furthermore, we showed that clonogenic forming ability was repressed by siMALAT1. The ability of transformed cells under anchorage-independent growth is important characteristic of carcinogenesis. This result supports that MALAT1 has contributed to tumorigenesis in gastric cancer cell.

In our study, dysregulation of MALAT1 was clearly shown to affect the epithelial-to-mesenchymal transition (EMT) through invasion and migration results. MALAT1 plays a role as a predictive marker for metastasis via the EMT pathway, and its most important regulatory motif is in its 3’ end (6918 nt- 8841 nt), which promotes invasion, migration, and cell proliferation [15]. One study has indicated that downregulation of MALAT1 inhibits metastasis through regulation of CTHRC1, CCT4, HMMR, and ROD1 [19]. Furthermore, we showed decreased migratory capacity and invasiveness following MALAT1 silencing, and found decreased expression of the mesenchymal markers, N-cadherin and snail, which are major factors that regulate the EMT in gastric cancer cells. In our gene expression array to investigate comprehensive mechanism of MALAT1 in gastric cancer, WNT/β-catenin signaling was affected by knock down of MALAT1. WNT/β-catenin signaling was considered to be correlated with tumor invasion in gastric cancer cell. The level of β-catenin in both mRNA and protein was down-regulated by knock down of MALAT1 in our study. The knock down of MALAT1 altered nuclear translocation of β-catenin. MALAT1 mediated-Wnt/β-catenin regulation has reported in oral squamous, esophageal squamous cells and colorectal cancer [27–29] but did not report in gastric cancer yet.

This study had some limitations. The first limitation is the small sample size. The small sample size makes difficult to determine exact impact of MALAT1 on patient’s survival in this study.

In summary, we found that deregulation of MALAT1 is involved in carcinogenesis and metastasis of gastric cancer. Although the exact mechanism thereof is not known, it is related to the alteration of RASSF6 and β-catenin caused by MALAT1. These findings suggest that gastric cancers share the same pathogenic pathway of MALAT1 in carcinogenesis as do lung, esophageal, brain, pancreatic, prostate, and liver cancer [17, 19, 30–32]. Our findings highlight the interaction among the lncRNA, MALAT1, RASSF6 and β-catenin during tumorigenesis and progression of gastric cancer.

## Conclusions

In our study, we have demonstrated that lncRNA MALAT1 regulates proliferation and metastasis in gastric cancer cells. Based on the microarray and gene expression results, we provide the evidence that the change of the phenotype by MALAT1 silencing is associated with the alteration of RASSF6 and β-catenin at transcriptional level. Consequently, lncRNA MALAT1 could be a new biomarker that being therapeutic target for diagnosis of gastric cancer.

## Additional file

Additional file 1: Cancer-related target gene expression by MALAT1 silencing. To analyze the relation among cancer-related target genes by siMALAT1, NanoString nCounter gene expression analysis was carried out. (XLSX 42 kb)

## Abbreviations

CCT4: Chaperonin Containing TCP1 Subunit 4
CTHRC1: Collagen Triple Helix Repeat Containing 1
EMT: Epithelial-to-mesenchymal transition
GSK-3β: Glycogen synthase kinase 3 beta
HMMR: Hyaluronan Mediated Motility Receptor
MALAT1: Metastasis associated lung adenocarcinoma transcript-1
MMP9: Matrix metallopeptidase 9
RASSF6: Ras association domain family (RASSF) 6
ROD1: Regulator Of Differentiation 1
SRSF1: Serine/arginine-rich splicing factor 1
ZEB1: Zinc finger E-box-binding homeobox 1
